# Association Between HALP Score and Atrial Fibrillation Recurrence After Radiofrequency Catheter Ablation

**DOI:** 10.1155/cdr/4538055

**Published:** 2026-05-11

**Authors:** Qing Yan, Yide Yuan, Yuyang Zhao, Lan Yang, Jiali Fan, Jiahong Xue, Qiangsun Zheng

**Affiliations:** ^1^ Department of Cardiovascular Medicine, The Second Affiliated Hospital of Xi′an Jiaotong University, Xi’an, Shaanxi, China

**Keywords:** AF recurrence, HALP score, radiofrequency catheter ablation

## Abstract

**Background:**

Radiofrequency catheter ablation (RFCA) is the primary treatment for atrial fibrillation (AF), but recurrence rates remain high. The hemoglobin, albumin, lymphocyte, and platelet (HALP) score reflects inflammation and nutrition, yet its prognostic value for AF recurrence remains unclear.

**Methods:**

We retrospectively reviewed 877 patients with AF undergoing first RFCA. Patients were divided into four quartiles (Q1–Q4) based on preoperative HALP scores. Recurrence‐free survival was estimated by Kaplan–Meier curves. Multivariate Cox models assessed the independent link between HALP scores and AF recurrence. Restricted cubic spline (RCS) analysis was conducted to explore the dose–response relationship. Model discrimination and reclassification were assessed using receiver operating characteristic (ROC) curve analysis, net reclassification improvement (NRI), and integrated discrimination improvement (IDI).

**Results:**

A total of 686 patients with AF (mean age: 63.4 ± 10.7 years; 55.5% male) were included in the final analysis. The risk of AF recurrence demonstrated an inverse trend across HALP score quartiles (log‐rank *p* < 0.001). After adjustment, patients in Q3 (HR = 0.45, 95% CI: 0.27–0.75) and Q4 (HR = 0.18, 95% CI: 0.08–0.38) had lower recurrence rates than those in Q1. RCS analysis revealed a linear inverse association between HALP score and the risk of AF recurrence. ROC analysis demonstrated that the HALP score alone exhibited significant discriminatory ability (AUC = 0.71, 95% CI: 0.66–0.75). Notably, incorporating the HALP score into a baseline model of 15 variables significantly improved model discrimination, increasing the AUC from 0.78 to 0.81 (*p* < 0.001). Furthermore, the addition of HALP score yielded significant improvements in reclassification (NRI = 0.19, IDI = 0.04; both *p* < 0.001).

**Conclusions:**

Preoperative HALP score is independently linked to AF recurrence after RFCA. Lower scores correspond to higher recurrence risk, suggesting its potential for enhanced risk stratification when combined with conventional clinical factors.

## 1. Introduction

Representing the most prevalent sustained arrhythmia encountered in clinical practice, atrial fibrillation (AF) poses a growing global challenge [1]. This condition correlates with a heightened probability of stroke, heart failure, and death, thereby generating significant financial burden on healthcare systems and society [2]. Radiofrequency catheter ablation (RFCA), grounded in circumferential pulmonary vein isolation (CPVI) [3], is currently recognized as the gold standard for rhythm control in symptomatic patients with AF refractory to antiarrhythmic drugs (AADs) [4, 5]. Despite advances in procedural techniques, arrhythmia recurrence occurs in 30%–50% of patients within the first year after ablation [6], underscoring the pressing need for improved risk stratification.

Multiple clinical and imaging‐based factors—such as the CHA2DS2‐VASc score [7], left atrial diameter (LAD) [8], and the extent of left atrial fibrosis [9]—have been associated with post‐ablation recurrence. Nevertheless, existing predictive models often lack sufficient discriminatory accuracy for routine clinical application [10]. Accordingly, there is a clear need to identify novel, readily accessible biomarkers that can enhance prognostic assessment.

Systemic inflammation and nutritional profiles are increasingly recognized as pivotal drivers of atrial remodeling and AF progression [11]. In this context, various combined inflammatory indices have emerged as effective strategies for predicting the likelihood of AF recurrence following catheter ablation [12–14], and accumulating evidence supports the role of baseline nutritional status in determining ablation outcomes [15–17]. The hemoglobin–albumin–lymphocyte–platelet (HALP) score is a novel composite index that integrates markers of inflammation, nutrition, and immunity function [18]. Existing literature has linked the HALP score to adverse cardiovascular outcomes, including mortality in patients with coronary artery disease [19] and a poor prognosis in AF populations [20]. Given its composite nature and biological plausibility, the HALP score may reflect the underlying substrate predisposing to AF recurrence.

However, its prognostic value specifically for arrhythmia recurrence after RFCA remains unexplored. This gap prompted us to evaluate the link between preoperative HALP scores and AF recurrence after RFCA.

## 2. Methods

### 2.1. Study Population

We conducted a retrospective cohort study at the Second Affiliated Hospital of Xi’an Jiaotong University. Eligibility for inclusion required patients to meet three conditions: (1) a confirmed diagnosis of nonvalvular AF; (2) undergoing their first RFCA; and (3) aged 18–80 years. A total of 877 individuals who satisfied these criteria were enrolled between September 2017 and May 2023. Exclusion criteria were as follows: (1) significant structural cardiac anomalies, including moderate‐to‐severe valvulopathy or cardiomyopathies (dilated/hypertrophic) (*n* = 30); (2) follow‐up duration < 1 year due to loss to follow‐up or death (*n* = 82); or (3) missing essential laboratory parameters during the index hospitalization (*n* = 79). Given the low proportion of missing data (< 10%), a complete‐case analysis was performed.

Ethical approval was obtained from the Institutional Ethics Committee of the Second Affiliated Hospital of Xi’an Jiaotong University (Approval No. 2022270), and the study was performed in compliance with the guidelines of the Declaration of Helsinki. The protocol mandated written informed consent from all subjects. In cases precluding written signature, verbal consent was formally documented following Ethics Committee guidelines.

### 2.2. Data Collection

Electronic medical records served as the source for extracting detailed demographic data, history of comorbidities, and results from laboratory and imaging tests. Key baseline variables included gender, age, body mass index (BMI), along with the occurrence rates of diabetes mellitus (DM), coronary heart disease (CHD), hypertension, and hyperlipidemia (characterized by high total cholesterol or triglyceride concentrations). Clinical variables spanning AF type and duration, ablation protocols, AADs regimens, and prior electrical cardioversion were also documented. Furthermore, key echocardiographic indices, specifically left ventricular ejection fraction (LVEF) and LAD, were captured for subsequent analysis.

Preoperative hematological parameters were obtained from routine laboratory tests performed after an overnight fast (≥ 8 h) immediately before ablation. These included lymphocyte count, platelet count, hemoglobin concentration, and serum albumin level. All samples were collected under standardized conditions to ensure accurate reflection of preoperative status and to minimize pre‐analytical variability.

### 2.3. HALP Score Calculation and Patient Grouping

The HALP score was derived based on the equation below: hemoglobin (g/L) × albumin (g/L) × lymphocyte count (/L)/platelet count (/L) [18].

Participants were divided into four groups (Q1–Q4) according to HALP score quartiles, with Q1 representing the lowest (≤ 25th percentile) and Q4 the highest (≥ 75th percentile).

### 2.4. Ablation Strategy and Postoperative Management

All RFCA procedures were performed by electrophysiologists with at least 5 years of experience. Detailed protocols have been described previously [21]. In brief, transesophageal echocardiography was performed before ablation to rule out left atrial or left atrial appendage thrombi. Procedures were conducted under sedation after an overnight fast. Following transseptal puncture via the right femoral vein, circular mapping catheters were placed in the left atrium, and three‐dimensional electroanatomical mapping was constructed using the EnSite or CARTO system.

The standardized ablation strategy was implemented as follows: CPVI served as the universal foundational strategy for all patients, regardless of AF type. For the majority of patients with paroxysmal AF, CPVI alone was deemed sufficient. In patients with persistent AF, CPVI was performed first, followed by electrical cardioversion if AF persisted. Adjunctive linear ablation was generally reserved for cases where AF organized into macro‐re‐entrant atrial tachycardia (AT) or was guided by specific underlying left atrial substrate. Specifically, in patients with a preserved left atrial substrate, CPVI remained the primary therapeutic endpoint. Conversely, in those exhibiting an abnormal substrate, further substrate modification was systematically implemented based on the anatomical and electrical properties of the atrium, including posterior box isolation, roof lines, mitral isthmus lines, and/or complex fractionated atrial electrogram (CFAE) ablation. Strict procedural endpoints were enforced: bidirectional CPVI block was confirmed by entrance and exit blocks, and linear lesions required bidirectional block verification via differential pacing. A standardized 20–30 min waiting period post‐isolation was observed, followed by an adenosine and isoproterenol challenge to unmask dormant conduction.

Contact force‐sensing catheters were utilized throughout the study period. Lesion durability was optimized using specific metrics: an Ablation Index (AI) of 350–400 (Biosense Webster) or Lesion Size Index (LSI) of 4.0–4.5 (Abbott systems) was targeted for the posterior wall, while an AI of 450–500 or LSI of 5.0 was applied to the anterior wall.

Following the procedure, all patients received AADs for the first 3 months to maintain sinus rhythm. Anticoagulation with rivaroxaban or warfarin (target international normalized ratio [INR] 2–3) was administered for at least 2 months. The duration of AADs and anticoagulant therapy during follow‐up was individualized based on patient status and in accordance with the 2024 ESC Guidelines for the management of AF [4].

### 2.5. Outcome Definitions and Follow‐Up After RFCA

AF recurrence after RFCA served as the primary endpoint. Specifically, AF recurrence was identified by the presence of any recorded atrial arrhythmia (AF, flutter, or tachycardia) lasting > 30 s, provided it was detected following the 3‐month blanking period [22].

Follow‐up visits were scheduled at 1, 3, 6, 9, and 12 months post‐procedure. Recurrence was confirmed by 12‐lead ECG or 24‐h Holter monitoring at each visit. Additional assessments were arranged if symptoms suggestive of recurrence occurred.

### 2.6. Statistical Analysis

Continuous variables are presented as mean ± SD, and categorical variables as frequencies (percentages). Group comparisons were performed using the chi‐square test or Fisher’s exact test for categorical data, and one‐way ANOVA or the Kruskal–Wallis test for continuous data, as appropriate. Recurrence‐free survival was estimated using Kaplan–Meier curves. Multivariable Cox proportional hazards models were used to assess the association between HALP score and AF recurrence. The proportional hazards assumption was verified using Schoenfeld residuals (global *p* = 0.88; all individual covariates *p* > 0.05). Receiver operating characteristic (ROC) curves were generated, and areas under the curve (AUCs) were compared using the DeLong test. The incremental prognostic value of adding the HALP score to the baseline model was evaluated using the integrated discrimination improvement (IDI) and net reclassification improvement (NRI). A restricted cubic spline (RCS) model with three knots was used to examine potential nonlinear relationships. All statistical analyses were performed using SPSS (v18.0) and R (v4.3.1). Statistical significance was defined as a two‐sided *p* value below 0.05.

## 3. Results

### 3.1. Participant Eligibility and Enrollment

**Figure 1:**
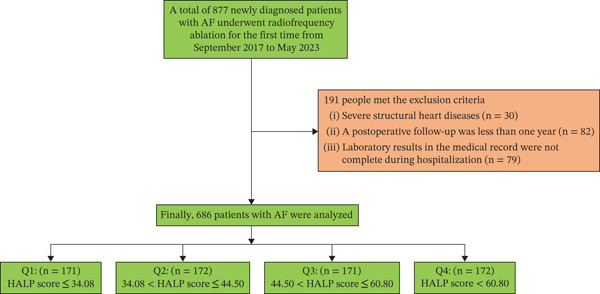
Flowchart of research subject screening.

Initially, 877 individuals met the inclusion criteria; of these, 191 were excluded according to the predefined exclusion protocol. The final analysis comprised 686 patients diagnosed with AF. Participants were stratified into four groups based on HALP score quartiles: Q1 (≤ 34.08, *n* = 171), Q2 (34.08–44.50, *n* = 172), Q3 (44.50–60.80, *n* = 171), and Q4 (>60.80, *n* = 172) (Figure 1).

### 3.2. Baseline Patient Characteristics

Among the 686 patients with AF (mean age: 63.4 ± 10.7 years; mean BMI: 24.3 ± 3.0 kg/m^2^; 55.5% male), 380 (55.4%) had paroxysmal AF. All patients underwent CPVI, and 100 underwent additional ablation, including left atrial roof line ablation (*n* = 18), cavotricuspid isthmus ablation (*n* = 49), and posterior wall box isolation (*n* = 33). Post‐procedural rhythm control was managed with oral amiodarone in 65.7% of patients. During the 1‐year follow‐up, 124 individuals (18.1%) experienced AF recurrence.

The distribution of baseline characteristics according to HALP score quartiles is shown in Table 1. Patients in the lowest quartile (Q1) were older, had longer AF duration, larger LAD, and higher recurrence rates (all *p* < 0.05). Sex, BMI, AF type, ablation strategy, electrical cardioversion usage, uric acid levels, LVEF, AAD utilization, and the prevalence of hyperlipidemia, hypertension, DM, and CHD were similarly distributed across groups (all *p* > 0.05).

**Table 1:** Comparison of baseline characteristics.

Variable	ALL (*n* = 686)	Q1 (*n* = 171)	Q2 (*n* = 172)	Q3 (*n* = 171)	Q4 (*n* = 172)	*p*
Male	381 (55.5%)	86 (50.2%)	93 (54.1%)	97 (56.7%)	105 (61.1%)	0.234
Age, years	63.4 ± 10.7	65.4 ± 10.6	64.6 ± 11.0	62.4 ± 10.2	61.0 ± 10.7	<0.001
BMI, kg/m^2^	24.3 ± 3.0	24.1 ± 3.2	24.5 ± 3.0	24.4 ± 3.3	24.0 ± 2.4	0.384
Hypertension	347 (50.6%)	99 (57.9%)	83 (48.3%)	85 (49.7%)	80 (46.5%)	0.156
Hyperlipidemia	69 (10.1%)	20 (11.7%)	12 (7.0%)	19 (11.1%)	18 (10.5%)	0.466
Diabetes	175 (25.5%)	48 (28.1%)	53 (30.8%)	34 (19.9%)	40 (23.3%)	0.092
CHD	166 (24.2%)	51 (29.8%)	33 (19.2%)	45 (26.3%)	37 (21.5%)	0.094
Paroxysmal AF	380 (55.4%)	89 (52.1%)	96 (55.8%)	94 (55.0%)	101 (58.7%)	0.666
Duration ≥ 2 years	355 (51.8%)	103 (60.2%)	94 (54.7%)	90 (52.6%)	68 (39.5%)	0.002
Electrical cardioversion	198 (28.9%)	53 (31.0%)	42 (24.4%)	54 (31.6%)	49 (28.5%)	0.447

Ablation strategy						0.359
CPVI	586 (85.4%)	139 (81.3%)	152 (88.4%)	145 (84.8%)	150 (87.2%)	
CPVI + right atrial isthmus line	49 (7.1%)	13 (7.6%)	11 (6.4%)	16 (9.4%)	9 (5.2%)	
CPVI + left atrial roof wall line	18 (2.6%)	6 (3.5%)	5 (2.9%)	4 (2.3%)	3 (1.8%)	
CPVI + left atrial posterior wall box	33 (4.8%)	13 (7.6%)	4 (2.3%)	6 (3.5%)	10 (5.8%)	

AADs						0.272
Amiodarone	451 (65.8%)	110 (64.3%)	112 (65.1%)	123 (71.9%)	106 (61.6%)	
Propafenone	20 (2.9%)	8 (4.7%)	3 (1.7%)	4 (2.3%)	5 (2.9%)	
Beta‐blockers	186 (27.1%)	48 (28.1%)	52 (30.3%)	35 (20.5%)	51 (29.7%)	
Amiodarone + Beta‐blockers	29 (4.2%)	5 (2.9%)	5 (2.9%)	9 (5.3%)	10 (5.8%)	
Uric acid, *μ*mol/L	317.6 ± 86.2	312.6 ± 95.7	310.3 ± 78.9	324.4 ± 87.0	323.3 ± 82.3	0.303
LVEF, %	62.9 ± 6.5	62.2 ± 6.1	62.9 ± 6.5	63.3 ± 6.8	63.1 ± 6.8	0.459
LAD, mm	37.1 ± 5.2	38.4 ± 5.5	36.7 ± 4.9	37.0 ± 5.0	36.4 ± 5.0	0.002
Recurrence	124 (18.1%)	53 (31.0%)	42 (24.4%)	21 (12.3%)	8 (4.7%)	<0.001

Abbreviations: AADs: antiarrhythmic drugs; AF: atrial fibrillation; BMI: body mass index; CHD: coronary heart disease; CPVI: circumpulmonary vein isolation; LAD: left atrial diameter; LVEF: left ventricular ejection fraction.

### 3.3. Clinical Outcomes After RFCA by HALP Quartile

**Figure 2:**
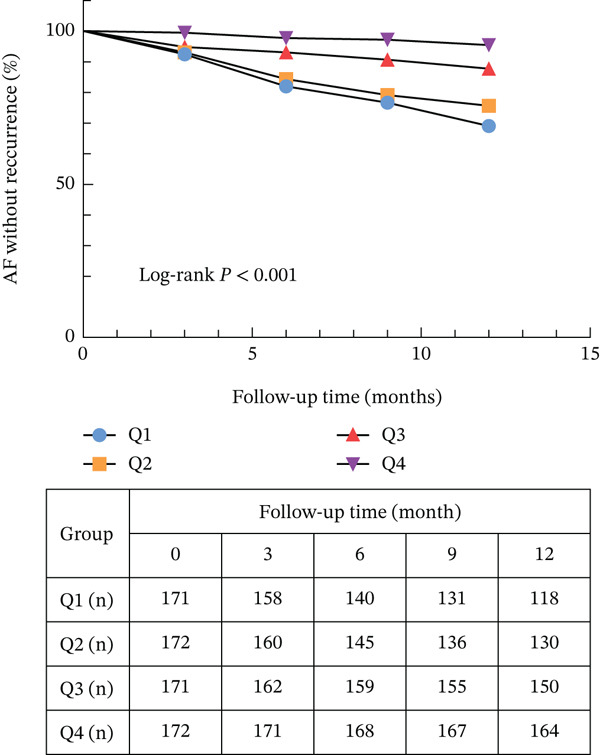
Kaplan–Meier survival curves for AF recurrence based on HALP score quartiles.

As shown in Figure 2, during the 1‐year follow‐up, 124 patients (18.1%) experienced AF recurrence, with event rates decreasing across HALP quartiles: 53 (31.0%) in Q1, 42 (24.4%) in Q2, 21 (12.3%) in Q3, and 8 (4.7%) in Q4. The incidence of recurrence differed significantly among groups (*p* < 0.001) (Table 1). Kaplan–Meier analysis revealed a stepwise reduction in cumulative AF recurrence risk with increasing HALP quartiles (log‐rank *p* < 0.001).

### 3.4. Adjusted Cox Analysis of HALP Score and Post‐RFCA AF Recurrence

In the unadjusted model (Model 1), patients in the Q3 (HR = 0.37, 95% CI: 0.22–0.61; *p* < 0.001) and Q4 (HR = 0.14, 95% CI: 0.06–0.28; *p* < 0.001) quartiles had significantly lower risks of AF recurrence compared with those in Q1. The Q2 group exhibited no significant association.

To mitigate the influence of confounding variables, we further developed three adjusted models by sequentially incorporating demographic and comorbidity variables (Model 2), laboratory parameters (Model 3), and clinical AF characteristics (Model 4). The associations remained robust across all adjusted models. Specifically, patients in the Q3 (Model 2: HR = 0.38, *p* < 0.001; Model 3: HR = 0.43, *p* = 0.001; Model 4: HR = 0.45, *p* = 0.002) and Q4 (Model 2: HR = 0.14, *p* < 0.001; Model 3: HR = 0.16, *p* < 0.001; Model 4: HR = 0.18, *p* < 0.001) groups exhibited a markedly reduced likelihood of AF recurrence relative to the Q1 cohort (Table 2).

**Table 2:** Results of COX regression analysis with multiple adjustments.

HALP score	Model 1		Model 2		Model 3		Model 4	
	HR (95% CI)	*p* value	HR (95% CI)	*p* value	HR (95% CI)	*p* value	HR (95% CI)	*p* value
Q1	—	—	—	—	—	—	—	—
Q2	0.78 (0.52–1.16)	0.221	0.83 (0.55–1.25)	0.365	0.93 (0.61–1.41)	0.740	0.91 (0.60–1.39)	0.673
Q3	0.37 (0.22–0.61)	<0.001	0.38 (0.23–0.64)	<0.001	0.43 (0.26–0.72)	0.001	0.45 (0.27–0.75)	0.002
Q4	0.14 (0.06–0.28)	<0.001	0.14 (0.07–0.29)	<0.001	0.16 (0.07–0.33)	<0.001	0.18 (0.08–0.38)	<0.001

*Note:* Model 1: No variables were adjusted. Model 2: Adjusted for gender, age, BMI, hypertension, hyperlipidemia, diabetes, and CHD. Model 3: Adjusted for uric acid, LVEF, LAD, and the variables in Model 2. Model 4: Adjusted for AF type, duration, electrical cardioversion, ablation strategy, AADs, and the variables in Model 3.

### 3.5. Incremental Discriminatory Value of the HALP Score

**Figure 3:**
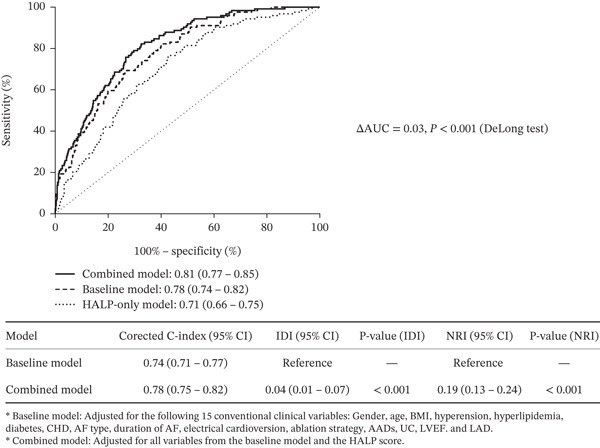
Comparison of the discrimination ability among the Baseline, HALP‐only, and Combined models.

To rigorously assess the additional prognostic information provided by the HALP score, we compared the ROC curves of a Baseline Model (defined by 15 established clinical and echocardiographic variables) with those of a Combined Model (Baseline Model plus HALP score) (Figure 3). Discriminatory ability was significantly enhanced by incorporating the HALP score. The Combined Model yielded an AUC of 0.81 (95% CI: 0.77–0.85), which was higher than the 0.78 (95% CI: 0.74–0.82) observed in the Baseline Model. The DeLong test confirmed the statistical significance of this improvement (*Δ*AUC = 0.03, *p* < 0.001). Consistent with the AUC findings, the optimism‐corrected C‐index also improved from 0.74 in the Baseline Model to 0.78 in the Combined Model, confirming the robustness of the added discriminatory ability. For reference, the HALP score alone demonstrated moderate discrimination with an AUC of 0.71 (95% CI: 0.66–0.75).

Beyond discrimination, reclassification metrics further confirmed the superior performance of the Combined Model. The IDI was 0.04 (95% CI: 0.01–0.07, *p* < 0.001), indicating an improvement in the average separation of predicted probabilities between events and non‐events. Moreover, the NRI was 0.19 (95% CI: 0.13–0.24; *p* < 0.001), indicating that incorporating the HALP score successfully reclassified approximately 19% of patients into more appropriate risk categories compared with the Baseline Model alone. Collectively, these findings demonstrate that the HALP score provides significant incremental prognostic information for AF recurrence prediction beyond traditional clinical parameters.

### 3.6. Nonlinear Association Between HALP Score and AF Recurrence Risk

**Figure 4:**
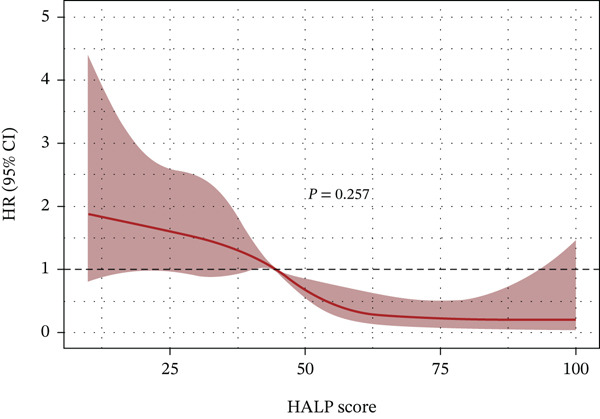
Restricted cubic spline plot of HALP score and risk of AF recurrence.

Figure 4 visualizes the RCS analysis, elucidating the dose–response pattern between baseline HALP levels and the probability of AF recurrence following RFCA. Statistical testing indicated no significant deviation from linearity (P for nonlinearity = 0.257), thereby confirming a predominantly linear inverse relationship. Specifically, the likelihood of AF recurrence exhibited a consistent downward trend as HALP scores incrementally increased across the observed range.

### 3.7. Subgroup Analysis

**Figure 5:**
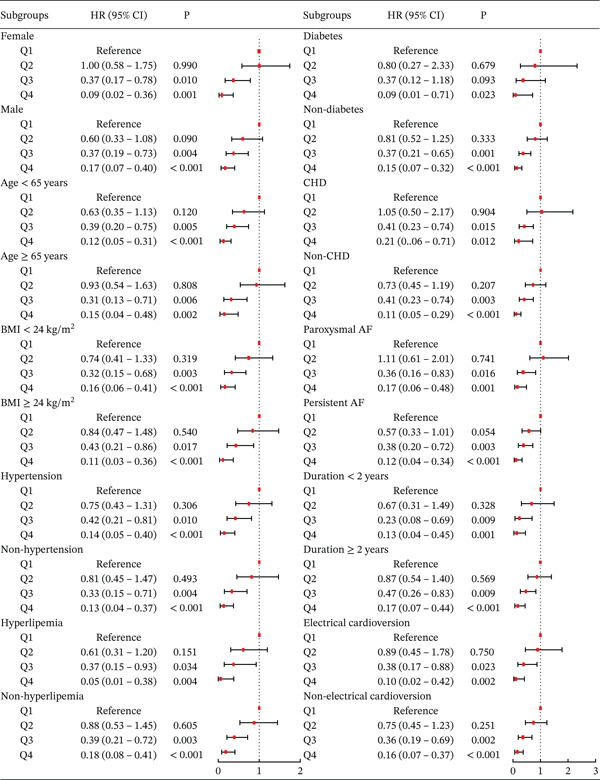
Main subgroup analysis of the relationship between HALP score and the risk of AF recurrence.

Subgroup analyses were conducted according to gender, age, BMI, hypertension, hyperlipidemia, DM, CHD, AF duration, AF type, and use of electrical cardioversion to examine the stability of the association (Figure 5). Across all subgroups, patients in the Q3 and Q4 groups had significantly lower risks of AF recurrence compared with Q1. In contrast, the Q2 group showed no significant risk reduction relative to Q1. Notably, the Q4 group consistently exhibited a lower recurrence risk than the Q3 group across all subgroups, reinforcing a graded protective effect of higher HALP scores.

## 4. Discussion

A significant relationship was observed between baseline HALP levels and the probability of AF recurrence after RFCA. Lower HALP scores were consistently associated with a higher likelihood of recurrence, even after adjustment for established clinical, laboratory, and procedural confounders, underscoring its role as a robust indicator of post‐ablation outcomes. Notably, incorporating the HALP score into the baseline model led to a marked improvement in discrimination. Furthermore, RCS analysis revealed a predominantly linear inverse relationship, with progressively lower risk as HALP scores increased. The consistency of these findings across all prespecified subgroups highlights the utility of the HALP score in stratifying risk among heterogeneous patient populations.

A growing body of evidence underscores the strong link between systemic inflammation, nutritional status, and the risk of AF recurrence after RFCA. With regard to systemic inflammation, a range of inflammatory biomarkers are now recognized for their prognostic utility in assessing post‐ablation recurrence [14, 23]. Regarding nutritional status, studies from China and Japan have demonstrated that patients with poorer baseline nutrition exhibit a higher likelihood of AF recurrence [16, 17]. Furthermore, a 2024 report highlighted the synergistic interplay between inflammation and nutrition in AF pathophysiology and outcomes [15]. Building on these observations, we evaluated the HALP score as a reliable indicator of AF recurrence following RFCA, owing to its unique combination of inflammatory and nutritional components. Unlike traditional single‐domain indices, the HALP score integrates hematopoiesis, immunity, inflammation, and nutritional status into a single composite metric. A notable inverse relationship was found between the HALP score and AF recurrence incidence, implying that diminished HALP values serve as a marker of elevated recurrence risk. These findings support the value of holistically assessing systemic host status to improve risk stratification and long‐term management in patients with AF.

While initially validated for prognostic stratification in oncological settings, the HALP score has recently emerged as a promising metric within cardiovascular medicine. Previous research has linked lower HALP scores to increased severity of peripheral artery disease [24]. In CHD, reduced HALP levels correlate with adverse clinical outcomes [19, 25–27], while also serving as a mortality marker in heart failure [28, 29]. Notably, among patients with AF, low HALP scores have been correlated with a higher need for mechanical ventilation and increased in‐hospital mortality [20]. Collectively, these findings suggest that reduced HALP scores reliably predict adverse events across a spectrum of cardiovascular diseases [30]. Our study extends this evidence by demonstrating that a low preoperative HALP score independently correlates with a greater risk of AF recurrence after RFCA, a relationship that remained robust across all predefined subgroups. These results imply that early identification of patients with compromised systemic status via the HALP score could facilitate targeted interventions to optimize clinical outcomes.

Of particular relevance, findings from Wang et al. [31] revealed that patients with persistent AF undergoing RFCA exhibited greater susceptibility to recurrence when presenting with lower HALP scores. In contrast, our study serves as a significant extension and robust validation of these prior findings within a broader AF population. We explicitly expand the scope of validation from persistent AF alone to encompass both paroxysmal and persistent forms. Beyond this expanded scope, we employed RCS analysis to rigorously characterize the dose–response relationship. Unlike studies assuming linearity without verification, our analysis confirmed a robust linear inverse association, ruling out significant nonlinear patterns. Additionally, we conducted comprehensive model comparisons using DeLong tests, IDI, and NRI to assess the incremental predictive value of adding the HALP score to the baseline model, further supporting its enhanced discrimination and reclassification ability. Furthermore, extensive and consistent subgroup analyses across diverse clinical characteristics demonstrated the stability of this association. In summary, rigorous statistical validation and the inclusion of diverse AF subtypes have substantially strengthened the utility of the HALP score for risk stratification, thereby underscoring the need to further investigate the underlying mechanisms driving this link.

The HALP score synthesizes markers of nutritional status, immunity, and inflammation into a unified metric, thereby capturing the complex interplay of systemic host factors. Specifically, low hemoglobin levels, indicative of anemia, may precipitate myocardial hypoxia and oxidative stress [32], thereby fostering an arrhythmogenic substrate through electrical and structural alterations that predispose patients to AF recurrence [33]. Concurrently, hypoalbuminemia serves as a dual marker of malnutrition and chronic inflammation; this pro‐inflammatory milieu can exacerbate atrial fibrosis and sustain the arrhythmic substrate [34, 35]. Furthermore, platelets act as pivotal mediators linking inflammation and coagulation [36]. In a hypercoagulable state, they may promote microthrombosis and endothelial dysfunction, further accelerating adverse atrial remodeling [37]. By integrating these interconnected pathophysiological pathways, the HALP score offers a holistic assessment of the patient’s systemic status, establishing itself as a robust predictor of clinical outcomes following RFCA.

Several limitations should be acknowledged in this study. First, the retrospective nature of this cohort study may have introduced potential selection and information bias. Second, the HALP score is susceptible to unmeasured comorbidities (e.g., chronic inflammatory or immune disorders), leaving the possibility of residual confounding despite adjustments for major clinical factors. Third, data on key covariates—including renal function (eGFR), liver function, active infection markers, and indicators of atrial remodeling (LAVI, BNP/NT‐proBNP)—were unavailable; their absence from the multivariable model may affect the precision of the HALP score’s independent predictive value. Fourth, HALP scores were assessed only at baseline without evaluation of dynamic changes during follow‐up. Fifth, the fixed 12‐month follow‐up limited the assessment of long‐term arrhythmia burden and late recurrences. Finally, the use of standard 12‐lead ECG and Holter monitoring may have underestimated the incidence of asymptomatic or subclinical AF episodes.

## 5. Conclusion

The preoperative HALP score is independently and inversely associated with AF recurrence after RFCA, exhibiting a linear relationship that is consistent across AF subtypes. As a comprehensive marker integrating inflammation, immunity, and nutrition, it offers a cost‐effective tool to enhance pre‐procedural risk stratification.

## Author Contributions

Qing Yan and Yide Yuan have contributed to the work equally and should be regarded as co‐first authors.

## Funding

This study was supported by the Key Research and Development Projects of Shaanxi Province (S2024‐YF‐YBSF‐0667 to Xue JH) and the Free Exploration Project of the Second Affiliated Hospital of Xi’an Jiaotong University (2020YJ(ZYTS)153 to Xue JH).

## Ethics Statement

The research was conducted ethically following the World Medical Association Declaration of Helsinki. This study was approved by the Ethics Review Board of the Second Affiliated Hospital of Xi’an Jiaotong University (NO. 2022270). All patients provided written informed consent before enrollment.

## Consent

The authors have nothing to report.

## Conflicts of Interest

The authors declare no conflicts of interest.

## Data Availability

The data that support the findings of this study are available from the corresponding author upon reasonable request.
